# Dr. Palmer on “Manual Training”

**Published:** 1893-02

**Authors:** 


### DR. PALMER ON "MANUAL TRAINING."

In the original department of the present number will be found an article by Dr. S. B. Palmer, of Syracuse, on the necessity of a change in the curriculum of dental colleges. The advice is most excellent, and, coming from one who is practically familiar with all forms of dental work, should be seriously considered by those institutions, if there be any, not up to his standard.

We are inclined to the opinion, however, that Dr. Palmer is not familiar with the work done in the best schools at the present time, but rather bases his conclusions on the past.

It was very true that during the "dark age" of dentistry, when vulcanite superseded other forms of plates, the art of working in metals was nearly lost to the profession. Students failed to receive instruction in the branches deemed so important in previous years. The result was that neither in private practice nor in college work was the true art of the mechanical dentist taught. A change came with the introduction of crown- and bridge-work, and from that period a return to metal working has become part of the teaching of all well-regulated colleges.

So far from this being confined to the last term of the three years' course, it is believed it is in all, as it certainly is in some of the institutions, continuous throughout the three years, and the student must be dull indeed who could regard his manual training as a farce at the end of his three years' course.

Much is still needed to make our colleges what they should be. The difficulties met with in teaching large bodies of men have not yet been overcome, but we believe that the time is not far distant when the highest present ideal will have been reached. The excellent suggestions given by our colleague and friend will then all have been incorporated in the curriculum.

